# Diaquabis­(thio­cyanato-κ*N*)bis[6-(4*H*-1,2,4-triazol-4-yl-κ*N* ^1^)pyridin-2-amine]­cadmium

**DOI:** 10.1107/S1600536812032473

**Published:** 2012-07-21

**Authors:** Yuan-Yuan Liu, Pan Yang, Bin Ding

**Affiliations:** a Tianjin Key Laboratory of Structure and Performance for Functional Molecule, Tianjin Normal University, Tianjin 300071, People’s Republic of China

## Abstract

In the title compound, [Cd(NCS)_2_(C_7_H_7_N_5_)_2_(H_2_O)_2_], the Cd^II^ cation lies on an inversion center and is coordinated by the N atoms of two thiocyanate anions, by N atoms of two 6-(4*H*-1,2,4-triazol-4-yl)pyridin-2-amine ligands and by the O atoms of two water molecules in a distorted N_4_O_2_ octa­hedral geometry. The dihedral angle between the triazole and pyridine rings is 23.15 (12)°. In the crystal, mol­ecules are linked by N—H⋯N and O—H⋯S hydrogen bonds. Offset π–π stacking between parallel pyridine rings of adjacent mol­ecules is also observed, the centroid–centroid distance being 3.6319 (14) Å.

## Related literature

For the preparation of the organic ligand, see: Gioia *et al.* (1988 ▶). For complexes with 4-3-pyridyl-1,2,4-triazole ligands, see: Moulton & Zaworotko (2001 ▶); Pan *et al.* (2001 ▶); Prior & Rosseinsky (2001 ▶); Ma *et al.* (2001 ▶); Ding *et al.* (2006 ▶); Liu *et al.* (2007 ▶).
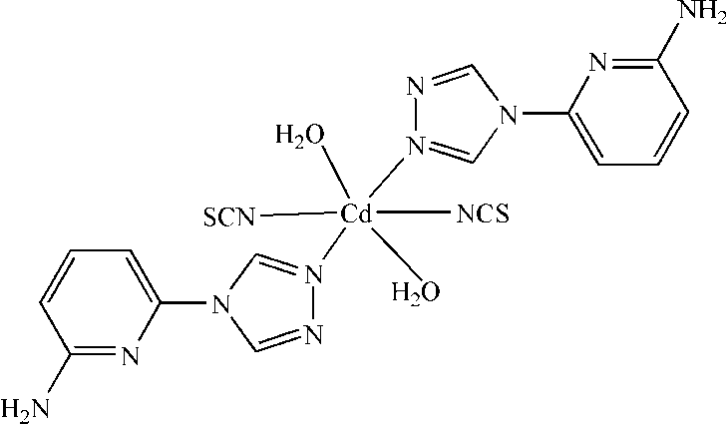


## Experimental

### 

#### Crystal data

[Cd(NCS)_2_(C_7_H_7_N_5_)_2_(H_2_O)_2_]
*M* *_r_* = 586.94
Triclinic, 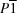

*a* = 7.5586 (15) Å
*b* = 7.5876 (15) Å
*c* = 11.311 (2) Å
α = 106.859 (2)°
β = 95.790 (2)°
γ = 110.883 (2)°
*V* = 564.7 (2) Å^3^
*Z* = 1
Mo *K*α radiation
μ = 1.19 mm^−1^
*T* = 293 K
0.20 × 0.16 × 0.12 mm

#### Data collection

Bruker APEXII CCD area-detector diffractometer
Absorption correction: multi-scan (*SADABS*; Bruker, 2001 ▶) *T*_min_ = 0.796, *T*_max_ = 0.870
3064 measured reflections
1957 independent reflections
1893 reflections with *I* > 2σ(*I*)
*R*_int_ = 0.010

#### Refinement

*R*[*F* ^2^ > 2σ(*F* ^2^)] = 0.017
*wR*(*F* ^2^) = 0.046
*S* = 1.05
1957 reflections
151 parameters
H-atom parameters constrained
Δρ_max_ = 0.31 e Å^−3^
Δρ_min_ = −0.22 e Å^−3^

### 

Data collection: *APEX2* (Bruker, 2007 ▶); cell refinement: *SAINT* (Bruker, 2007 ▶); data reduction: *SAINT*; program(s) used to solve structure: *SHELXS97* (Sheldrick, 2008 ▶); program(s) used to refine structure: *SHELXL97* (Sheldrick, 2008 ▶); molecular graphics: *DIAMOND* (Brandenburg, 1999 ▶); software used to prepare material for publication: *SHELXL97*.

## Supplementary Material

Crystal structure: contains datablock(s) global, I. DOI: 10.1107/S1600536812032473/xu5596sup1.cif

Structure factors: contains datablock(s) I. DOI: 10.1107/S1600536812032473/xu5596Isup2.hkl

Additional supplementary materials:  crystallographic information; 3D view; checkCIF report

## Figures and Tables

**Table 1:** Selected bond lengths (Å)

Cd1—N1	2.2785 (16)
Cd1—N6	2.3146 (19)
Cd1—O1	2.3501 (15)

**Table 2:** Hydrogen-bond geometry (Å, °)

*D*—H⋯*A*	*D*—H	H⋯*A*	*D*⋯*A*	*D*—H⋯*A*
O1—H1*A*⋯S1^ii^	0.85	2.51	3.3468 (17)	168
O1—H1*B*⋯S1^iii^	0.85	2.51	3.3575 (17)	172
N5—H5*A*⋯N2^iv^	0.86	2.23	3.080 (2)	169
N5—H5*B*⋯N6^v^	0.86	2.57	3.422 (3)	170

Symmetry codes: (ii) 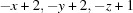; (iii) 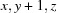; (iv) 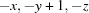; (v) 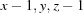.

**Fig. 1.**
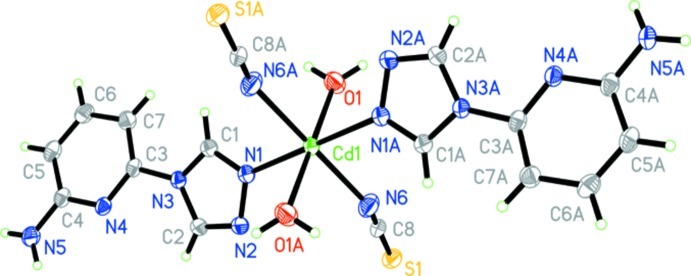
The molecular structure of (I) with atom labels and 30% probability dis- placement ellipsoids for non-H atoms..

**Fig. 2.**
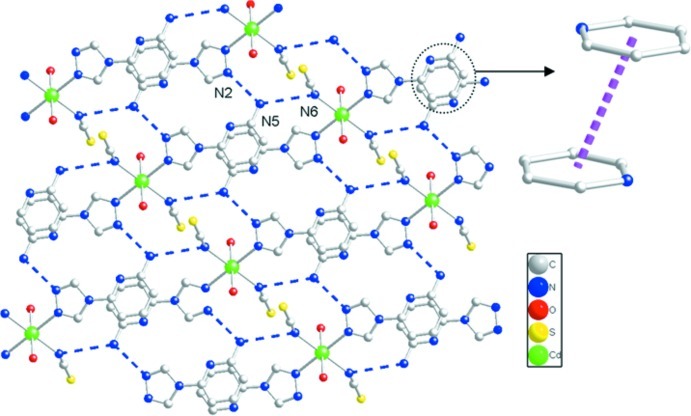
The two-dimensional supra-molecular network stabilized via N···N hydrogen bonds and offset π···π stacking interactions, Blue lines represent N—H···N hydrogen bonds.

**Fig. 3.**
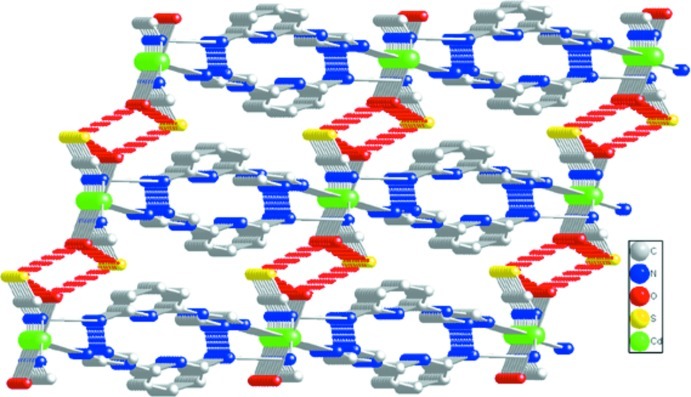
The three-dimensional supramolecular packing architecture of (I). Red lines represent O—H···S hydrogen bonds and Blue lines represent N—H···N hydrogen bonds.

## supplementary crystallographic information

### Comment

Recently, considerable efforts have been devoted to crystal engineering of supramolecular architecture sustained by coordination covalent bonding, hydrogen bonding or some molecular interaction and their combination owing to their fascinating structural diversity and potential application in design of porous materials with novel inclusion or reactivity properties and in supramolecular devices such as sensor and indicator (Moulton *et al.*, 2001; Pan *et al.*, 2001; Ma *et al.*, 2001; Prior *et al.*, 2001). Our interest is toexploit the coordination chemistry of 1,2,4-triazole and its derivatives together with their potential application in material science (Liu *et al.*, 2007; Ding *et al.*, 2006).

In the report, the mono-nuclear Cadmium(II) complex was obtained *via* the reaction of 2-amino-6-(4-triazoyl)pyridine, NH_4_NCS and corresponding Cadmium(II) salts. A view of the coordination compound [Cd(II)*L*_2_(NCS)_2_(H_2_O)_2_] is shown in Figure 1. Single crystal X-ray diffraction analysis reveals that the the Cadmium(II) atom is six-coordinated by two pyridine nitrogen atoms, two NCS nitrogen atoms and two aqua oxygen atoms forming N_4_O_2_ donor set. Bond distances of Cd—N and Cd—O(Cd(1)—N(1):2.2785 (16) \%A; Cd(1)—N(6):2.3146 (19) \%A; Cd(1)—O(1):2.3500 (15) \%A) are listed. The coordination geometry around the Cadmium(II) center in the molecular lattice lie in the inversion center and can be described as the Octahedral geometry.

L is mono-dentate terminal ligand coordinated *via* its pyridine nitrogen atoms. The weak N···N interactions between *L* triazole rings (N—H···N, 3.080 (2) and 3.422 (3) \%A) between *L* triazole rings can be observed, The offset π···π stacking interactions between two neighboring pyridine rings are also important for the assembly of the supra-molecular structure,the ring centroid-centroid distance being 3.632 (3) Å. As shown in Figure 2, A two-dimensional supra-molecular network can be observed stablized *via* N···N interactions and π···π stacking interactions.

Further the non-classic O—H···S hydrogen bonds (O—H···S, 3.346 (8) and 3.357 (5) \%A) also can be observed, which further assembly these two-dimensional supramolecular network to form a three-dimensional supra-molecular structure. The three-dimensional packing architecture in the unit cell of the complex is shown in Figure 3.

### Experimental

The organic ligand *L* was prepared according to the previously reported literature methods (Gioia *et al.*, 1988). A mixture of CdBr_2_ (27.2 mg, 0.1 mmol), NH_4_NCS (7.6 mg, 0.1 mmol), *L* (14.6 mg, 0.1 mmol) and water (10 ml) was stirred for 5 h and filtered. Suitable single crystals for X-ray diffraction study were obtained after a few days, yield 23% (based on Cd(II) salts). Anal. Calc. for C_16_H_18_CdN_12_O_2_S_2_: C, 32.74%; H, 3.09%; N, 28.63%. Found: C, 32.86%; H, 3.18%; N, 28.74%. FT-IR (KBr):. 3404w, 3281w, 3135w, 2969w, 2918w, 2069 s, 1625 s, 1524*m*, 1405 s, 1247*m*, 1096*m*, 1017*m*, 792w, 676w,618w, 529w cm^-1^.

### Refinement

The H atoms of the aromatic rings were placed at calculated positions, with C—H = 0.93 \%A and O—H = 0.85 \%A. All H atoms were assigned fixed isotropic displacement parameters, with *U*ιso(H) = 1.2*_U_*_eq_(C) or 1.5*_U_*_eq_(O).

### Crystal data

**Table:**

[Cd(NCS)_2_(C_7_H_7_N_5_)_2_(H_2_O)_2_]	*Z* = 1
*M**_r_* = 586.94	*F*(000) = 294
Triclinic, *P*1	*D*_x_ = 1.726 Mg m^−^^3^
Hall symbol: -P 1	Mo *K*α radiation, λ = 0.71073 Å
*a* = 7.5586 (15) Å	Cell parameters from 2419 reflections
*b* = 7.5876 (15) Å	θ = 3.0–27.8°
*c* = 11.311 (2) Å	µ = 1.19 mm^−^^1^
α = 106.859 (2)°	*T* = 293 K
β = 95.790 (2)°	Block, colorless
γ = 110.883 (2)°	0.20 × 0.16 × 0.12 mm
*V* = 564.7 (2) Å^3^	

### Data collection

**Table:**

Bruker APEXII CCD area-detector diffractometer	1957 independent reflections
Radiation source: fine-focus sealed tube	1893 reflections with *I* > 2σ(*I*)
Graphite monochromator	*R*_int_ = 0.010
phi and ω scans	θ_max_ = 25.0°, θ_min_ = 1.9°
Absorption correction: multi-scan (*SADABS*; Bruker, 2001)	*h* = −8→8
*T*_min_ = 0.796, *T*_max_ = 0.870	*k* = −9→8
3064 measured reflections	*l* = −11→13

### Refinement

**Table:**

Refinement on *F*^2^	0 restraints
Least-squares matrix: full	H-atom parameters constrained
*R*[*F*^2^ > 2σ(*F*^2^)] = 0.017	*w* = 1/[σ^2^(*F*_o_^2^) + (0.0263*P*)^2^ + 0.1431*P*] where *P* = (*F*_o_^2^ + 2*F*_c_^2^)/3
*wR*(*F*^2^) = 0.046	(Δ/σ)_max_ = 0.001
*S* = 1.05	Δρ_max_ = 0.31 e Å^−^^3^
1957 reflections	Δρ_min_ = −0.22 e Å^−^^3^
151 parameters	

### Special details

**Table:**

Geometry. All e.s.d.'s (except the e.s.d. in the dihedral angle between two l.s. planes) are estimated using the full covariance matrix. The cell e.s.d.'s are taken into account individually in the estimation of e.s.d.'s in distances, angles and torsion angles; correlations between e.s.d.'s in cell parameters are only used when they are defined by crystal symmetry. An approximate (isotropic) treatment of cell e.s.d.'s is used for estimating e.s.d.'s involving l.s. planes.
Refinement. Refinement of *F*^2^ against ALL reflections. The weighted *R*-factor *wR* and goodness of fit *S* are based on *F*^2^, conventional *R*-factors *R* are based on *F*, with *F* set to zero for negative *F*^2^. The threshold expression of *F*^2^ > σ(*F*^2^) is used only for calculating *R*-factors(gt) *etc*. and is not relevant to the choice of reflections for refinement. *R*-factors based on *F*^2^ are statistically about twice as large as those based on *F*, and *R*- factors based on ALL data will be even larger.

### Fractional atomic coordinates and isotropic or equivalent isotropic displacement parameters (Å^2^)

**Table:**

	*x*	*y*	*z*	*U*_iso_*/*U*_eq_	
Cd1	0.5000	1.0000	0.5000	0.03514 (8)	
S1	0.76959 (8)	0.51242 (8)	0.30994 (5)	0.04950 (14)	
O1	0.7956 (2)	1.2313 (2)	0.48659 (14)	0.0558 (4)	
H1A	0.8961	1.2929	0.5473	0.084*	
H1B	0.7767	1.2993	0.4425	0.084*	
N1	0.3864 (2)	0.8993 (2)	0.28661 (14)	0.0389 (4)	
N2	0.3014 (2)	0.6986 (2)	0.20991 (15)	0.0444 (4)	
N3	0.2754 (2)	0.8835 (2)	0.09699 (14)	0.0333 (3)	
N4	0.0703 (2)	0.7895 (2)	−0.09637 (14)	0.0362 (3)	
N5	−0.1479 (3)	0.6777 (3)	−0.28550 (16)	0.0536 (5)	
H5A	−0.2021	0.5648	−0.2745	0.064*	
H5B	−0.1931	0.6948	−0.3522	0.064*	
N6	0.6234 (3)	0.7555 (3)	0.46977 (19)	0.0591 (5)	
C1	0.3697 (3)	1.0051 (3)	0.21725 (17)	0.0381 (4)	
H1	0.4162	1.1448	0.2465	0.046*	
C2	0.2365 (3)	0.6948 (3)	0.09812 (18)	0.0419 (4)	
H2	0.1716	0.5777	0.0277	0.050*	
C3	0.2201 (3)	0.9376 (3)	−0.00828 (16)	0.0342 (4)	
C4	0.0087 (3)	0.8284 (3)	−0.19785 (17)	0.0392 (4)	
C5	0.0995 (3)	1.0167 (3)	−0.21024 (19)	0.0451 (5)	
H5	0.0560	1.0407	−0.2815	0.054*	
C6	0.2529 (3)	1.1644 (3)	−0.1160 (2)	0.0469 (5)	
H6	0.3138	1.2902	−0.1227	0.056*	
C7	0.3184 (3)	1.1270 (3)	−0.00964 (19)	0.0428 (4)	
H7	0.4223	1.2246	0.0560	0.051*	
C8	0.6818 (3)	0.6545 (3)	0.40295 (19)	0.0419 (4)	

### Atomic displacement parameters (Å^2^)

**Table:**

	*U*^11^	*U*^22^	*U*^33^	*U*^12^	*U*^13^	*U*^23^
Cd1	0.03953 (12)	0.03434 (12)	0.02935 (11)	0.01460 (8)	0.00083 (8)	0.01088 (8)
S1	0.0553 (3)	0.0447 (3)	0.0527 (3)	0.0247 (2)	0.0111 (2)	0.0178 (2)
O1	0.0496 (8)	0.0550 (9)	0.0509 (9)	0.0073 (7)	0.0026 (7)	0.0226 (7)
N1	0.0424 (9)	0.0367 (8)	0.0337 (8)	0.0146 (7)	0.0009 (7)	0.0113 (7)
N2	0.0504 (9)	0.0345 (8)	0.0394 (9)	0.0105 (7)	−0.0045 (7)	0.0137 (7)
N3	0.0345 (8)	0.0358 (8)	0.0298 (7)	0.0149 (6)	0.0040 (6)	0.0121 (6)
N4	0.0438 (8)	0.0380 (8)	0.0302 (8)	0.0205 (7)	0.0055 (7)	0.0128 (6)
N5	0.0694 (12)	0.0486 (10)	0.0373 (9)	0.0225 (9)	−0.0090 (8)	0.0163 (8)
N6	0.0773 (13)	0.0569 (11)	0.0539 (11)	0.0424 (11)	0.0085 (10)	0.0178 (9)
C1	0.0420 (10)	0.0348 (9)	0.0351 (10)	0.0157 (8)	0.0034 (8)	0.0108 (8)
C2	0.0456 (10)	0.0352 (10)	0.0369 (10)	0.0126 (8)	−0.0027 (8)	0.0100 (8)
C3	0.0387 (9)	0.0424 (10)	0.0309 (9)	0.0233 (8)	0.0118 (7)	0.0162 (8)
C4	0.0492 (11)	0.0472 (11)	0.0312 (9)	0.0287 (9)	0.0115 (8)	0.0156 (8)
C5	0.0564 (12)	0.0572 (12)	0.0383 (10)	0.0315 (10)	0.0171 (9)	0.0276 (9)
C6	0.0522 (12)	0.0485 (12)	0.0539 (12)	0.0232 (10)	0.0211 (10)	0.0310 (10)
C7	0.0414 (10)	0.0451 (11)	0.0431 (11)	0.0155 (9)	0.0098 (9)	0.0197 (9)
C8	0.0450 (10)	0.0379 (10)	0.0432 (11)	0.0172 (9)	−0.0017 (9)	0.0181 (9)

### Geometric parameters (Å, º)

**Table:**

Cd1—N1	2.2785 (16)	N4—C3	1.324 (2)
Cd1—N1^i^	2.2786 (16)	N4—C4	1.345 (2)
Cd1—N6	2.3146 (19)	N5—C4	1.354 (3)
Cd1—N6^i^	2.3146 (19)	N5—H5A	0.8600
Cd1—O1^i^	2.3500 (15)	N5—H5B	0.8600
Cd1—O1	2.3501 (15)	N6—C8	1.152 (3)
S1—C8	1.644 (2)	C1—H1	0.9300
O1—H1A	0.8501	C2—H2	0.9300
O1—H1B	0.8501	C3—C7	1.368 (3)
N1—C1	1.301 (2)	C4—C5	1.403 (3)
N1—N2	1.381 (2)	C5—C6	1.366 (3)
N2—C2	1.297 (3)	C5—H5	0.9300
N3—C1	1.348 (2)	C6—C7	1.396 (3)
N3—C2	1.359 (2)	C6—H6	0.9300
N3—C3	1.438 (2)	C7—H7	0.9300
			
N1—Cd1—N1^i^	180.00 (8)	C4—N5—H5A	120.0
N1—Cd1—N6	90.53 (6)	C4—N5—H5B	120.0
N1^i^—Cd1—N6	89.47 (6)	H5A—N5—H5B	120.0
N1—Cd1—N6^i^	89.47 (6)	C8—N6—Cd1	147.28 (17)
N1^i^—Cd1—N6^i^	90.53 (6)	N1—C1—N3	110.42 (17)
N6—Cd1—N6^i^	179.999 (1)	N1—C1—H1	124.8
N1—Cd1—O1^i^	89.76 (6)	N3—C1—H1	124.8
N1^i^—Cd1—O1^i^	90.24 (6)	N2—C2—N3	111.46 (17)
N6—Cd1—O1^i^	89.56 (7)	N2—C2—H2	124.3
N6^i^—Cd1—O1^i^	90.45 (7)	N3—C2—H2	124.3
N1—Cd1—O1	90.24 (5)	N4—C3—C7	126.62 (17)
N1^i^—Cd1—O1	89.76 (6)	N4—C3—N3	113.17 (15)
N6—Cd1—O1	90.45 (7)	C7—C3—N3	120.21 (17)
N6^i^—Cd1—O1	89.55 (7)	N4—C4—N5	116.29 (17)
O1^i^—Cd1—O1	180.0	N4—C4—C5	121.60 (18)
Cd1—O1—H1A	123.2	N5—C4—C5	122.09 (17)
Cd1—O1—H1B	111.3	C6—C5—C4	118.96 (18)
H1A—O1—H1B	115.7	C6—C5—H5	120.5
C1—N1—N2	107.86 (15)	C4—C5—H5	120.5
C1—N1—Cd1	129.58 (13)	C5—C6—C7	120.27 (19)
N2—N1—Cd1	122.24 (11)	C5—C6—H6	119.9
C2—N2—N1	105.99 (16)	C7—C6—H6	119.9
C1—N3—C2	104.27 (15)	C3—C7—C6	115.68 (18)
C1—N3—C3	128.48 (15)	C3—C7—H7	122.2
C2—N3—C3	127.20 (15)	C6—C7—H7	122.2
C3—N4—C4	116.86 (16)	N6—C8—S1	178.77 (19)
			
N1^i^—Cd1—N1—C1	168 (6)	C3—N3—C1—N1	177.31 (16)
N6—Cd1—N1—C1	−146.87 (17)	N1—N2—C2—N3	0.3 (2)
N6^i^—Cd1—N1—C1	33.13 (17)	C1—N3—C2—N2	0.0 (2)
O1^i^—Cd1—N1—C1	123.58 (17)	C3—N3—C2—N2	−177.66 (17)
O1—Cd1—N1—C1	−56.42 (17)	C4—N4—C3—C7	0.3 (3)
N1^i^—Cd1—N1—N2	−5 (6)	C4—N4—C3—N3	179.64 (15)
N6—Cd1—N1—N2	40.36 (15)	C1—N3—C3—N4	−155.32 (17)
N6^i^—Cd1—N1—N2	−139.64 (15)	C2—N3—C3—N4	21.7 (2)
O1^i^—Cd1—N1—N2	−49.19 (14)	C1—N3—C3—C7	24.1 (3)
O1—Cd1—N1—N2	130.81 (14)	C2—N3—C3—C7	−158.83 (19)
C1—N1—N2—C2	−0.5 (2)	C3—N4—C4—N5	−178.20 (17)
Cd1—N1—N2—C2	173.68 (13)	C3—N4—C4—C5	0.4 (3)
N1—Cd1—N6—C8	26.0 (3)	N4—C4—C5—C6	−0.7 (3)
N1^i^—Cd1—N6—C8	−154.0 (3)	N5—C4—C5—C6	177.76 (19)
N6^i^—Cd1—N6—C8	−19 (5)	C4—C5—C6—C7	0.5 (3)
O1^i^—Cd1—N6—C8	115.8 (3)	N4—C3—C7—C6	−0.5 (3)
O1—Cd1—N6—C8	−64.2 (3)	N3—C3—C7—C6	−179.85 (16)
N2—N1—C1—N3	0.5 (2)	C5—C6—C7—C3	0.1 (3)
Cd1—N1—C1—N3	−173.11 (11)	Cd1—N6—C8—S1	123 (9)
C2—N3—C1—N1	−0.3 (2)		

Symmetry code: (i) −*x*+1, −*y*+2, −*z*+1.

### Hydrogen-bond geometry (Å, º)

**Table:**

*D*—H···*A*	*D*—H	H···*A*	*D*···*A*	*D*—H···*A*
O1—H1*A*···S1^ii^	0.85	2.51	3.3468 (17)	168
O1—H1*B*···S1^iii^	0.85	2.51	3.3575 (17)	172
N5—H5*A*···N2^iv^	0.86	2.23	3.080 (2)	169
N5—H5*B*···N6^v^	0.86	2.57	3.422 (3)	170

Symmetry codes: (ii) −*x*+2, −*y*+2, −*z*+1; (iii) *x*, *y*+1, *z*; (iv) −*x*, −*y*+1, −*z*; (v) *x*−1, *y*, *z*−1.

## References

[bb1] Brandenburg, K. (1999). *DIAMOND* Crystal Impact GbR, Bonn, Germany.

[bb2] Bruker (2001). *SADABS* Bruker AXS Inc., Madison, Wisconsin, USA.

[bb3] Bruker (2007). *APEX2* and *SAINT* Bruker AXS Inc., Madison, Wisconsin, USA.

[bb4] Ding, B., Yi, L., Wang, Y., Cheng, P., Liao, D.-Z., Yan, S.-P., Jiang, Z.-H., Song, H.-B. & Wang, H.-G. (2006). *Dalton Trans.* pp. 65–675. 10.1039/b508332j 16429169

[bb5] Gioia, G. L., Bonati, F., Cingolania, A., Leonesia, D. & Lorenzottia, A. (1988). *Synth. React. Inorg. Met. Org. Chem.* **18**, 535–550.

[bb6] Liu, Y.-Y., Huang, Y.-Q., Shi, W., Cheng, P., Liao, D.-Z. & Yan, S.-P. (2007). *Cryst. Growth Des* **7**, 1483–1489.

[bb7] Ma, B.-Q., Gao, S., Sun, H.-L. & Xu, G.-X. (2001). *J. Chem. Soc. Dalton Trans.* pp. 130–133.

[bb8] Moulton, B. & Zaworotko, M. J. (2001). *Chem. Rev.* **101**, 1629–1658. 10.1021/cr9900432 11709994

[bb9] Pan, L., Ching, N., Huang, X.-Y. & Li, J. (2001). *Chem. Eur. J.* **7**, 4431–4437. 10.1002/1521-3765(20011015)7:20<4431::aid-chem4431>3.0.co;2-p 11695677

[bb10] Prior, T. J. & Rosseinsky, M. J. (2001). *Chem. Commun.* pp. 1222–1223.

[bb11] Sheldrick, G. M. (2008). *Acta Cryst.* A**64**, 112–122. 10.1107/S0108767307043930 18156677

